# The regulatory roles of lncRNAs in the process of breast cancer invasion and metastasis

**DOI:** 10.1042/BSR20180772

**Published:** 2018-09-28

**Authors:** Siying Zhou, Yunjie He, Sujin Yang, Jiahua Hu, Qian Zhang, Wei Chen, Hanzi Xu, Heda Zhang, Shanliang Zhong, Jianhua Zhao, Jinhai Tang

**Affiliations:** 1 The First Clinical Medical College, Nanjing University of Chinese Medicine, Xianlin Road 138, Nanjing 210023, P.R. China; 2 The First Clinical School of Nanjing Medical University, Nanjing 210029, P.R. China; 3 The Fourth Clinical School of Nanjing Medical University, Nanjing 210029, P.R. China; 4 Center of Clinical Laboratory Science, Jiangsu Cancer Hospital & Jiangsu Institute of Cancer Research & The Affiliated Cancer Hospital of Nanjing Medical University, Baiziting 42, Nanjing 210029, P.R. China; 5 Department of Head and Neck Surgery, Jiangsu Cancer Hospital & Jiangsu Institute of Cancer Research & The Affiliated Cancer Hospital of Nanjing Medical University, Baiziting 42, Nanjing 210029, P.R. China; 6 Department of Radiotherapy, Jiangsu Cancer Hospital & Jiangsu Institute of Cancer Research & The Affiliated Cancer Hospital of Nanjing Medical University, Baiziting 42, Nanjing 210029, P.R. China; 7 Department of General Surgery, School of Medicine, Southeast University, 87 Ding Jia Qiao, Nanjing 210009, P.R. China; 8 Department of General Surgery, the First Affiliated Hospital with Nanjing Medical University, Nanjing 210029, P.R. China

**Keywords:** Breast cancer (BC), Invasion, Long non-coding RNAs (lncRNAs), Metastasis

## Abstract

Breast cancer (BC) is the most common cancer and principal cause of death among females worldwide. Invasion and metastasis are major causes which influence the survival and prognosis of BC. Therefore, to understand the molecule mechanism underlying invasion and metastasis is paramount for developing strategies to improve survival and prognosis in BC patients. Recent studies have reported that long non-coding RNAs (lncRNAs) play critical roles in the regulation of BC invasion and metastasis through a variety of molecule mechanisms that endow cells with an aggressive phenotype. In this article, we focused on the function of lncRNAs on BC invasion and metastasis through participating in epithelial-to-mesenchymal transition, strengthening cancer stem cells generation, serving as competing endogenous lncRNAs, influencing multiple signaling pathways as well as regulating expressions of invasion–metastasis related factors, including cells adhesion molecules, extracellular matrix, and matrix metallo-proteinases. The published work described has provided a better understanding of the mechanisms underpinning the contribution of lncRNAs to BC invasion and metastasis, which may lay the foundation for the development of new strategies to prevent BC invasion and metastasis.

## Introduction

Breast cancer (BC), as the most common malignant tumor among women, is one of the leading causes of cancer deaths worldwide. In 2017, approximately 252710 new cases of invasive BC and 40610 BC deaths are expected to occur among US women [1]. BC invasion and metastasis are the main causes of BC-related deaths. Bone, lung, brain, and liver are the primary target sites of BC metastasis [2]. BC metastasis is the spread of cancer cells to tissues and organs beyond where the tumor originated and the formation of new tumors which may eventually result in the death of most BC patients [3]. At least half of the cancer patients already present clinically detectable metastatic disease when the time of cancer diagnosis [4]. A higher number of cancer patients might also have micrometastases that is beyond conventional detection techniques. Thus, cancer metastasis is the most threatening event in cancer patients [5]. BC invasion and metastasis as intricate process means that cancer cells escape from the primary cancer and penetrate the blood circulation [6]. The process involves both the selection of traits that are advantageous to cancer cells and the concomitant recruitment of traits in the tumor stroma that accommodate invasion by metastatic cells [7,8]. The course of BC invasion and metastasis entails a series of molecules such as cells adhesion molecules (CAMs), extracellular matrix (ECMs), and matrix metallo-proteinases (MMPs). It also involves the biological progresses including epithelial-to-mesenchymal transition (EMT) and cancer stem cells (CSCs) formation that cooperate on the formation of secondary tumors in distant organs [2,9]. Long non-coding RNAs (lncRNAs) are a novel class of RNA transcripts that are longer than 200 nucleotides (nt) in length without protein-coding capacity. The major functions of lncRNAs include: (1) participating in chromosome rearrangement and histone modification, (2) transcribing and interfering, (3) stabilizing mRNA, and (4) modifying alternative splicing sequence [10]. Recent studies have shown that lncRNAs exerted critically roles in multiple cancer biological processes, including carcinogenesis, apoptosis, differentiation, proliferation, invasion as well as metastasis [11]. Accumulating evidence has shown that ectopic expression of lncRNAs served as carcinogenic factors or tumor suppressors in BC invasion and metastasis [12,13]. In this review, we will sum up the precise mechanism of lncRNAs function on BC invasion and metastasis and reveal the clinical significance of dysregulated lncRNAs in BC metastasis. Knowledge obtained from this review could assist in the development of new strategies to treat or prevent the metastatic BC.

## LncRNAs participate in process of BC invasion and metastasis

**Figure 1:**
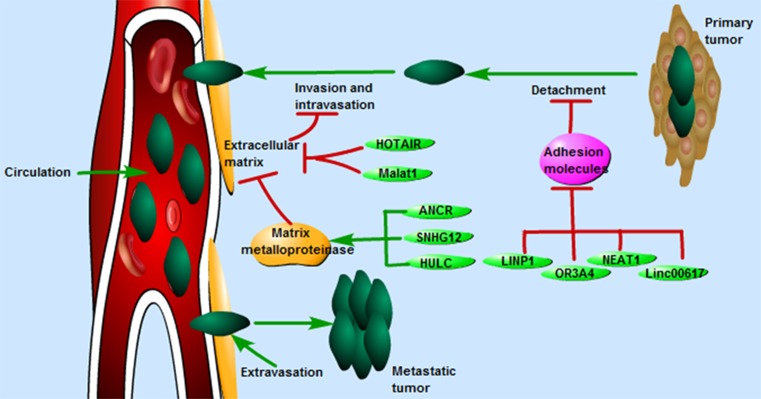
The process of BC invasion and metastasis includes detachment, invasion, intravasation, circulation, and extravasation LncRNAs such as NEAT1, linc00617, OR3A4, LINP1, HOTAIR, Malat1, SNHG12, HULC, ANCR, and BANCR were reported to participate the process of BC invasion and metastasis by regulating different molecules including CAMs, ECMs, and MMPs.

The process of BC invasion and metastasis is complex, which incorporates molecular factors, multiple cells, and stages. Some cancer cells detach from primary tumor through the repression of CAMs and the disruption of intercellular adhesion (detachment), followed by these cells invading through the ECMs and breaking down of ECMs (invasion), thus entering the circulation (intravasation). From this point, these cancer cells move away from the primary tumor and circulate in the blood circulation. Some cancer cells will adopt a process to leave the blood circulation (extravasation), in which cells adhere and penetrate the blood vessel again, eventually develop a secondary tumor at the other site [14,15] (Figure 1).

### CAMs

CAMs include immunoglobulin superfamily, cadherins, integrins, and selectins which provide essential links between the extracellular environment and the intracellular signaling pathways. Thus, CAMs play key roles in cells behaviors such as differentiation, apoptosis, invasion, and even metastasis [16,17]. Among them, E-cadherin (E-cad) as the most well-studied member of the CAMs displays a crucial type of cell–cell adhesion to hold cancer cells tight together. An increasing number of studies have shown that down-regulation of E-cad by lncRNAs could decrease the strength of cancer cellular adhesion, resulting in the increase in cellular motility. This in turn may allow cells to cross the CAMs and invade surrounding blood vessels in various cancers. For example, overexpression of lncRNA SPRY4-IT1 could increase in vitro motility of esophageal squamous cells carcinoma cells via decrease in E-cad [18]. In colon cancer, lncRNA activated by TGF-β (lncRNA-ATB) mediated E-cad repression may promote the progression of cancer and predicts poor prognosis [19]. In another study, lncRNA H19 levels were remarkably increased in bladder cancer tissues, and up-regulated H19 promoted bladder cancer cells migration by down-regulation of E-cad [20]. In BC, lncRNAs such as NEAT1, linc00617, OR3A4, and LINP1 were up-regulated in BC samples and significantly promoted the invasion and metastasis capacity in BC cells through decreasing the expression of E-cad [21–24].

### ECMs

ECMs such as collagen, fibrinogen, laminin, fibronectin, and vitronectin provide structural and biochemical support to the surrounding cells. ECMs are essential for many cellular processes, including development, migration, and proliferation [25–28]. LncRNAs play key roles in the complex dynamics of cancer cells invasion and metastasis through the regulation of ECMs. The elevation of lncRNA HOTAIR mediated invasion and metastasis of BC. HOTAIR expression exhibited robust induction in laminin-rich ECMs through the canonical ECM signaling pathway, namely integrins and Src kinase [29]. In addition, lncRNA Malat1 knockdown could also increase adhesion and inhibit migration of BC cells through the up-regulation of Tenascin Xb (Tnxb), Tnxb as an ECM protein that has been shown to have anti-metastatic property [12].

### MMPs

MMPs act important roles in tissue remodeling associated with various biology processes such as morphogenesis, angiogenesis, tissue repair, and metastasis. MMPs are also important components of cells invasion capable of degrading a range of ECMs proteins allowing cancer cells to migrate and invade [30–32]. Recent researches confirmed that lncRNAs regulate tumor invasion and metastasis partly because of their abilities to influence the expressions of MMPs. Such as, lncRNA SNHG12 was strongly up-regulated in Triple-negative BC (TNBC). Moreover, SNHG12 could promote BC cells migration by increasing expression of MMP13 [33]. In addition, HULC was another up-regulated lncRNA in TNBC tissues and cells lines. The increased HULC effectively promoted TNBC cells metastasis through regulating the expressions of MMP-2 and MMP-9 [34]. MMP-2 and MMP-9 could degrade basement-membrane collagen at the site of local invasion, thereby promoting cancer cells invasion and metastasis [35]. The expression of MMP-2 and MMP-9 could also be regulated by lncRNA ANCR. ANCR could act as a negative regulator of metastasis in BC cells through repressing MMP-9 and MMP-2 expression [36]. The expression level of lncRNA BANCR in BC tissues was significantly increased. After BANCR knockdown in BC MCF-7 cells, the cells invasion and metastasis capacity was significantly inhibited by decreasing expression of MMP-2 and MMP-9 [37].

Collectively, these findings revealed that lncRNAs such as NEAT1, linc00617, OR3A4, LINP1, HOTAIR, Malat1, SNHG12, HULC, ANCR, and BANCR affected BC cells invasion and metastasis through regulating different molecules including CAMs, ECMs, and MMPs (Figure 1). Therefore, lncRNAs may serve as therapeutic targets for BC, particularly in patients with BC metastasis.

## LncRNAs participate in BC metastasis via affecting EMT

EMT is a progression of cellular plasticity critical for BC cells migration and metastasis. It is characterized by the combined an increased expression of mesenchymal markers, such as vimentin, N-cadherin, fibronectin, and loss of epithelial cells junction proteins, including E-cad, claudins, α-catenin, ZO-1, and occludin [38–40]. Transforming growth factor-β (TGF-β) signaling pathway is a key regulator of various cancer biology, including cancer cells migration and invasion. TGF-β signaling pathway acts as an important regulator for EMT process through influencing the expressions of EMT-associated genes such as Zinc finger E-box-binding homeobox (ZEB), C/EBPb, RhoA, E-cad, vimentin, SNAIL, etc. [6].

Accumulating evidence shows that lncRNAs act crucial roles on EMT to influence BC metastasis. For instance, lncRNA ANCR could restrain the TGF-β1-induced EMT by inhibiting the phosphorylation of runt-related transcription factor 2 (RUNX2). On the contrary, TGF-β could decrease ANCR expression through decreasing the histone acetylation at ANCR promoter [41]. Moreover, ANCR could facilitate the ubiquitination and degradation of EZH2 (the Enhancer of Zeste Homolog 2), hence inhibit the invasion and metastasis of BC cells. The forced expression of EZH2 (an EMT inducer) increased the vimentin expression and decreased the E-cad expression [36]. LncRNA CCAT2 also plays an essential role in BC tumorigenesis, growth, and metastasis. The level of CCAT2 in BC tissues was significantly increased compared with adjacent normal tissues. The increased amount of CCAT2 promoted the proliferation, invasion, and migration by significantly reinforcing the expressions of TGF-β and Smad2 in BC cells [42]. Moreover, lncRNA-ATB was the most remarkably up-regulated lncRNA in trastuzumab-resistant (TR) SKBR-3 cells. The up-regulated lncRNA-ATB could promote invasion–metastasis cascade in BC by competitively binding miR-200c, increasing ZNF-217 and ZEB1, and then inducing EMT [43]. What is more, the increased ZNF217 could further increase the activity of TGF-β signaling by transcriptionally activating Smad 2/3 and ZEB [44]. Furthermore, lncRNA HOXA cluster antisense RNA2 (HOXA-AS2) was also up-regulated in BC tissues and cells lines. High level of HOXA-AS2 could inhibit miR-520c-3p, then release its target, TGF-β receptor 2 (TβR2), thus promote the migration and invasion of BC cells [45]. Zou et al. found that lncRNA TP73-AS1 promoted BC cells invasion and migration through competing with ZEB1 3’UTR for miR-200a binding. Moreover, ZEB1 could activate the expression of TP73-AS1 via binding to the promoter region of TP73-AS1. TP73-AS1/miR-200a/ ZEB1 as a regulating loop in BC cells could promote BC cells invasion and migration [46]. The recent study also demonstrated that linc-ROR as a competing endogenous RNA to mir-205 could prevent the degradation of ZEB1/2 [47]. LncRNA MEG3 was significantly down-regulated in BC tissues compared with adjacent normal tissues. MEG3 could inhibit BC cells proliferation and invasion capacities by down-regulating the levels of ZEB1/2 [20].

**Figure 2:**
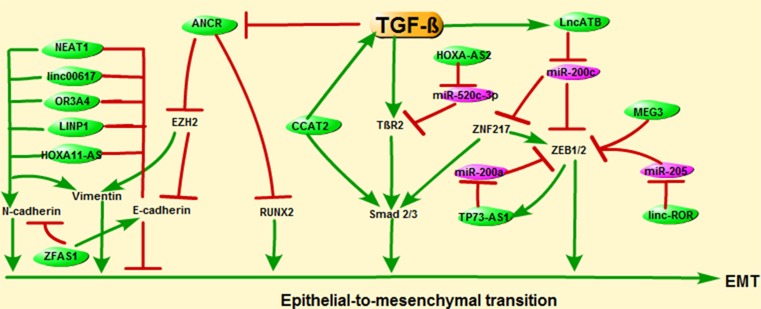
EMT is a progression of cellular plasticity critical for BC cells migration and metastasis LncRNAs such as ANCR, CCAT2, lncRNA-ATB, and HOXA-AS2 participate in BC invasion and metastasis through TGF-β-induced EMT. In addition, EMT-associated genes such as ZEB1/2, E-cad, N-cad, and vimentin were reported to be regulated by lncRNAs TP73-AS1, linc-ROR, MEG3, HOXA11-AS, NEAT1, linc00617, OR3A4, and LINP1.

The regulatory roles of lncRNAs on EMT are based on loss of expression of the E-cad and increase in expression of mesenchymal markers, such as N-cadherin, vimentin, which collectively result in the acquisition of a characteristic mesenchymal and migratory phenotype of BC cells. LncRNAs such as HOXA11-AS, NEAT1, linc00617, OR3A4, and LINP1 were reported to be related to EMT via decreasing the E-cad and increasing the mesenchymal markers vimentin and N-cadherin [21–24,48]. On the contrary, the up-regulation of lncRNA ZFAS1 could inhibit BC cells migration and invasion by regulating EMT. As expected, overexpression of ZFAS1 increased the expression of the epithelial marker E-cad while decreasing the expression of the mesenchymal markers N-cadherin and vimentin in MDA-MB-231 cells (Figure 2) [49].

Together, these results suggest that lncRNAs such as ANCR, CCAT2, lncRNA-ATB, and HOXA-AS2 participate in BC invasion and metastasis through TGF-β-induced EMT. In addition, EMT-associated genes such as ZEB1/2, E-cad, N-cad, and vimentin were reported to be regulated by lncRNAs TP73-AS1, linc-ROR, MEG3, HOXA11-AS, NEAT1, linc00617, OR3A4, and LINP1 (Figure 2). Thus, lncRNAs are involved in invasion and metastasis of BC cells by promoting EMT, providing potential therapeutic targets for BC.

## LncRNAs influence BC metastasis by regulating CSCs generation

CSCs are a subpopulation of cancer cells with self-renewal capacity and high invasion and migration capacity as well as limitless proliferative potential [50,51]. Recent studies illustrate a direct link between lncRNAs and stemness of cancer cells. LncRNAs have emerged as important new players in the regulation of CSCs stemness acquisition and maintenance [52,53]. Recently, lncRNA linc00617 was reported to promote BC invasion and metastasis through increasing the percentage of the stem cells phenotype CD44(+)/CD24(−) subpopulation cells. Furthermore, the up-regulated linc00617 promoted generation of stem-like cells and elevation of self-renewal ability in BC cells [22]. Also, the increased lncRNA linc-ROR was confirmed to affect the acquisition of CSC-like phenotype and the self-renewal capacity in BC cells. Linc-ROR promoted metastasis of BC cells by enhancing the ability to form multiple large-sized mammospheres [47]. What is more, lncRNA-Hh which was transcriptionally regulated by Twist could directly target GAS1 to stimulate the activation of hedgehog pathway. The activated hedgehog pathway increased the expression of GLI1, SOX2, and OCT4 to play a regulatory role in CSCs maintenance of BC cells [54]. In addition, lncRNA MALAT1 played a critical role in the acquisition of CSC-like phenotype of BC cells. Lysine-specific demethylase 5B protein (KDM5B) could enhance the maintenance of aggressive BC cells malignant phenotype via modulation of lncRNA MALAT1 activity [55].

Thus, these results suggest that lncRNAs including linc00617, linc-ROR, lncRNA-Hh, and MALAT1 play important roles in CSCs generation to increase invasion and migration capacity of BC cells. They may be explored as a prognostic and diagnostic molecule in BC patients with metastasis.

## LncRNAs serve as ceRNAs to influence BC metastasis

Dysregulated miRNAs participate in multiple biological processes such as apoptosis, proliferation, invasion, and metastasis by repressing translation or induce degradation of their target mRNAs [56,57]. LncRNAs act crucial roles on BC metastasis partly via sponging to miRNAs or regulating the expression of miRNAs. For instance, NEAT1 could promote BC cells growth, migration, and invasion by inhibiting miR-448 and up-regulating ZEB1. NEAT1 could serve as a competing endogenous lncRNA (ceRNA) to modulate ZEB1 by sponging miR-448 in BC [58]. In addition, miR-218 was reported to be another direct target of NEAT1. NEAT1 promoted BC cells invasion by negatively regulating expression of miR-218 [59]. It was also found that down-regulation of NEAT1 could also inhibit EMT program of BC cells through miR-211/high mobility group AT-hook 2 (HMGA2) axis and there was a reciprocal repression between NEAT1 and miR-211 [60]. HMGA2 has been reported to promote the action of transcriptional enhancers through binding to AT-rich regions in DNA and altering chromatin architecture. It is highly expressed in most cancers, including ovary, pancreas, lung, and BC, suggesting that HMGA2 could promote tumor progression in BC [61,62]. HMGA2 was identified as a target of miR-20a-5p, which significantly induced carcinogenesis of BC. MiR-20a-5p as a target of lncRNA HOTAIR had a negative correlation with HMGA2. HOTAIR affected BC cells growth, metastasis, and apoptosis via the miR-20a-5p/HMGA2 axis in BC cells [63]. Moreover, the pro-metastatic role of lncRNA MALAT1 on BC migration and invasion was mediated by miR-129-5p. MiR-129-5p is a direct inhibitory target of MALAT1 and its expression has an inverse correlation with MALAT1 [64]. Another study showed that the ectopic lncRNA SNHG15 was correlated with TNM stage, lymph node metastasis, and survival in BC patients. Increased SNHG15 significantly reinforced the abilities of BC cells migration and invasion. SNHG15 could act as a competing endogenous RNA to sponge miR-211-3p [65]. In MCF-7 cells, repression of HOST2 was remarkably elevated in the expression of miRNA let-7b. Down-regulation of HOST2 could decrease BC cells motility, migration, and invasion by inhibiting let-7b in BC patients [66]. On the contrary, lncRNA XIST was significantly down-regulated in BC tissues and cells lines. Overexpression of XIST remarkably inhibited BC cells growth, migration, and invasion via sponging to miR-155 in BC. Moreover, caudal-type homeobox 1 (CDX1) was identified as a direct target of miR-155 and miR-155/CDX1 rescued the effects of XIST in BC cells [67]. BC migration and invasion could also be regulated by lncRNA SUMO1P3. Further study confirmed that SUMO1P3 functioned as an oncogenic lncRNA via binding to miR-320a, which had been identified as a tumor suppressor in many cancers [68]. LncRNA cancer susceptibility candidate 2 (CASC2) has been demonstrated to be a tumor suppressor in several types of cancer [69]. In the present study, the expression level of CASC2 was significantly decreased in BC tissues compared with adjacent normal tissues. The up-regulated CASC2 decreased the viability, migration, and invasion, and promoted apoptosis of BC cells through acted as a ceRNA for miR-96-5p, while miR-96-5p overexpression could increase BC cells viability, migration, and invasion by repressing the expression of its target gene, synoviolin (SYVN1) [70] (Table 1).

**Table 1:** LncRNAs participate in BC metastasis by sponging to miRNA

LncRNA	Expression	Sponging miRNA	Function	Reference
NEAT1	Up	miR-448, miR-218, miR-211	NEAT1 facilitated cell growth and invasion via negatively regulating miR-218, as well as by regulating miR-211/HMGA2 axis and miR-448/ZEB1 axis in BC.	[58–60]
HOTAIR	Up	miR-20a-5p	HOTAIR affected BC cell growth, metastasis, and apoptosis via the miR-20a-5p/HMGA2 axis.	[63]
MALAT1	Up	miR-129-5p	MALAT1 promoted triple-negative BC invasion via targeting miR-129-5p.	[64]
SNHG15	Up	miR-211-3p	SNHG15 promoted BC cell migration and invasion by sponging miR-211-3p.	[65]
HOST2	Up	let-7b	HOST2 decreased BC cell motility, migration, and invasion by inhibiting let-7b.	[66]
XIST	Down	miR-155	XIST inhibited BC cell growth, migration, and invasion via miR-155/CDX1 axis.	[67]
SUMO1P3	Up	miR-320a	SUMO1P3 facilitated BC progression by negatively regulating miR-320a.	[68]
CASC2	Down	miR-96-5p	CASC2 inhibited the growth and metastasis of BC through the miR-96-5p/SYVN1 axis.	[70]

## LncRNAs regulate multiple signaling pathways in BC metastasis

Changes in the multiple signaling pathways directing their regulation can lead to the pathological process of BC cells invasion and metastasis. For example, lncRNA LINP1 could promote BC cells metastasis and influence the expression of EMT-related markers. P53 overexpression inhibited BC cells migration partly by decreasing LINP1 expression. P53 is a regulator of LINP1 while increased LINP1 could in turn attenuate the anti-metastatic effects of p53 [24]. P53 signaling pathway has a key role in the negative regulation of tumor angiogenesis, migration, and cells motility [71,72]. P21 as a classical downstream gene of the p53 signaling pathway was also associated various cancers progression, including the migration and invasion of BC [73–76]. LncRNA lincIN acted as a new regulator of BC metastasis at both transcriptional and translational levels. The up-regulated lincIN could repress p21 protein expression by inhibiting translation of p21 [77]. The lncRNA associated with BC brain metastases (Lnc-BM) is prognostic of the progression of brain metastasis in BC patients. In preclinical murine models, elevated Lnc-BM expression drove BC brain metastases, while depletion of Lnc-BM effectively treated BC brain metastases. Lnc-BM could increase JAK2 kinase activity to mediate IL-6- and oncostatin M-triggered STAT3 phosphorylation. In BC cells, Lnc-BM could trigger the activation of downstream signaling pathway of STAT3 that included the proteins CCL2 and ICAM1. The increased CCL2 and ICAM1 could attract macrophages and mediate vascular co-option in the brain, respectively. The attracted macrophages in turn released oncostatin M and IL-6, thereby further activating the Lnc-BM/JAK2/STAT3 pathway and enhancing BC brain metastases [78]. LncRNA CCAT2 was dysregulated in several cancers such as gastric cancer, colon cancer, and BC. Increased CCAT2 was associated with tumor metastasis, growth, and chromosomal instability [79]. The high expression level of CCAT2 could increase proliferation and invasion BC cells by activating the Wnt signaling pathway. CCAT2 could enhance the expression of downstream genes of Wnt/β-catenin signaling pathway, such as CCND1 and c-myc. In addition, the increased CCAT2 expression could activate the Wnt/β-catenin signaling pathway, and combination of increased CCAT2 and c-myc could synergistically improve the activation of Wnt signaling pathway [80]. In addition, DKK1, an inhibitor of Wnt signaling pathway, could inhibit the migration and invasion of BC [81,82]. Up-regulation of lncRNA NBAT1 could significantly increase the expression of DKK1 to inhibit migration and invasion of BC [83].

## Clinical significance of lncRNAs in BC metastasis

Accumulating evidence suggested that lncRNAs were abnormal expressed in various cancers and associated with cancer metastasis [84,85]. Dysregulated lncRNAs are also significantly related with multiple clinicopathological characteristics of BC, such as histological grade, ERBB2 expression, steroid-receptor expression, tumor size, TNM stage, and even lymph-node-metastasis (LNM) [12,86]. The expression of lncRNA H19 decreased significantly in adjacent normal tissues compared with BC tissues and plasma (*P*<0.05), and plasma levels of lncRNA H19 were significantly correlated with LNM (*P*=0.006) [87]. LncRNA TUG1 expression was significantly decreased in BC tissues compared with controls, and low TUG1 expression was significantly correlated with LNM (*P*=0.044) [88]. LncRNA FGF14 antisense RNA 2 (FGF14-AS2) was significantly restrained in BC tissues compared with adjacent normal tissues. Down-regulation of lncRNA FGF14-AS2 was correlated with more lymph node metastasis (*P*=0.000) [89]. BC patients with increased expressions of AC010729.1 and RP11-482H16.1 had a shorter metastasis-free survival (MFS) compared with patients with low expressions of AC010729.1 and RP11-482H16.1. Further analysis showed that AC010729.1 and RP11-482H16.1 could be useful prognostic markers to predict metastatic risk in BC patients [90]. LncRNA MALAT1 expressions were remarkably increased in 85.9% (67/78) of cancer tissues compared with adjacent normal tissues (*P*<0.01). Furthermore, the increased lncRNA MALAT1 in BC tissues was significantly associated with LNM (*P*=0.037) [91]. In matched metastatic and primary cancers, the expression of lncRNA HOTAIR increased in the metastatic carcinomas and its expression was a predictor of poor survival of BC patients [92]. Compared with BC tissues, lncRNA Z38 was down-regulated in normal breast tissues. High level of Z38 was significantly related to TNM stage and LNM [93]. The level of MEG3 was significantly decreased in BC tissues compared with adjacent normal tissues. Down-regulated MEG3 was significantly associated with TNM stage and LNM in BC patients [20]. The level of lncRNA OR3A4 was up-regulated in BC tissues compared with the adjacent normal tissues. The expression level of OR3A4 was found to be associated with LNM, differentiation grade, HER-2/neu status, and TNM stage [23]. LncRNA LINC01296 was aberrantly expressed in both BC tissue samples and cells. Increased LINC01296 was positive correlated with larger tumor size, positive LNM, and advanced TNM stage of patients with BC. Additionally, Cox regression analysis confirmed that LINC01296 could be an independent prognostic indicator for patients with BC [94]. LncRNA p10247 expression level in BC tissues was significantly up-regulated. More importantly, the overexpressed p10247 was associated with the development and progression of BC. The up-regulated expression of p10247 was significantly associated with progression of clinical stage (*P*=0.045) and LNM (*P*=0.039) [95] (Table 2).

**Table 2:** Clinical significance of LncRNAs in BC metastasis

LncRNA	Expression	Clinical significance	Reference
MEG3	Down	Down-regulated MEG3 was significantly associated with TNM stage and LNM in BC patients.	[20]
OR3A4	Up	The expression level of OR3A4 was found to be associated with LNM, differentiation grade, HER-2/neu status, and TNM stage.	[23]
H19	Up	Plasma levels of lncRNA H19 were significantly correlated with LNM.	[87]
TUG1	Down	Low TUG1 expression was significantly correlated with LNM.	[88]
FGF14-AS2	Down	Down-regulation of lncRNA FGF14-AS2 was correlated with more lymph node metastasis.	[89]
AC010729.1	–	Increased expressions of AC010729.1 and RP11-482H16.1 had a shorter MFS compared with patients with low expressions of AC010729.1 and RP11-482H16.1.	[90]
RP11-482H16.1			
AC010729.1			
RP11-482H16.1			
MALAT1	Up	Up-regulated MALAT1 was related to LNM.	[91]
HOTAIR	Up	HOTAIR increased in the metastatic carcinomas and its expression in BC was a predictor of poor survival.	[92]
Z38	Up	High level of Z38 was significantly related to advanced TNM stage and LNM.	[93]
LINC01296	Up	Increased LINC01296 was positive correlated with larger tumor size, positive LNM, and advanced TNM stage of patients with BC.	[94]
p10247	Up	The up-regulated expression of p10247 was significantly associated with progression of clinical stage and LNM.	[95]

## Prospects

The emerging evidence demonstrated that lncRNAs played key roles in various biological processes of BC. However, only a few lncRNAs have been well characterized in BC invasion and metastasis. While surgery can successfully treat primary BC, invasion and metastasis have proven to be a largely insurmountable challenge [96–98]. One primary challenge in BC patients is that metastasis has already occurred prior to diagnosis. Thus, it is essential to find useful prognostic markers to predict metastatic risk in BC patients, which may allow BC patients to choose an individualized treatment. LncRNAs are stable expression in tissue and plasma which may provide markers for a noninvasive and rapid diagnosis for metastatic BC. However, it is well known that the molecular mechanisms of lncRNAs are complicated, including chromatin remodeling, cells cycle control, genetic imprinting, splicing regulation, translational regulation as well as mRNA decay. The detailed molecular mechanisms underlying the function of lncRNAs on BC invasion and metastasis are still unresolved mysteries. Thus, future researches are needed to reveal the underlying mechanisms of dysregulated lncRNAs influence BC metastasis. In the wake of developments in molecular biologic techniques, such as next-generation sequencing and microarray, perhaps the mystery of lncRNAs in BC invasion and metastasis will be unveiled soon. An improved understanding of the BC invasion and metastasis regulated by lncRNAs could assist the development of new strategies to treat or prevent metastatic BC.

## Conclusion

In this review, we focused on the roles of lncRNAs as regulators during BC invasion and metastasis. Dysregulated lncRNAs participate in BC invasion and metastasis through participating in EMT transition, strengthening CSCs generation, serving as ceRNAs, influencing multiple signaling pathways as well as regulating expressions of CAMs, ECMs, and MMPs. We summarized the function and molecular mechanism of lncRNAs in BC invasion and metastasis as well as discussing the clinical significance of lncRNAs in BC staging and LNM. LncRNAs may act as pro-metastatic or anti-metastatic roles in BC. Through a clear understanding of the mechanism of lncRNAs in BC invasion and metastasis, lncRNAs could be promising therapeutic targets and novel molecular biomarkers for metastatic BC.

## Abbreviations

BC: breast cancer
CAM: cells adhesion molecule
CASC2: cancer susceptibility candidate 2
CDX1: caudal-type homeobox 1
ceRNA: competing endogenous lncRNA
CSC: cancer stem cell
E-cad: E-cadherin
ECM: extracellular matrix
EMT: epithelial-to-mesenchymal transition
EZH2: enhancer of Zeste Homolog 2
HMGA2: high mobility group AT-hook 2
lncRNA: long non-coding RNA
LNM: lymph-node-metastasis
MFS: metastasis-free survival
MMP: matrix metallo-proteinase
RUNX2: runt-related transcription factor 2
SYVN1: synoviolin
TβR2: TGF-β receptor 2
TGF-β: transforming growth factor-β
Tnxb: Tenascin Xb
TR: trastuzumab-resistant
ZEB: Zinc finger E-box-binding homeobox
